# Dynamically reorganized chromatin is the key for the reprogramming of somatic cells to pluripotent cells

**DOI:** 10.1038/srep17691

**Published:** 2015-12-07

**Authors:** Kaimeng Huang, Xiaobai Zhang, Jiejun Shi, Mingze Yao, Jiannan Lin, Jiao Li, He Liu, Huanhuan Li, Guang Shi, Zhibin Wang, Biliang Zhang, Jiekai Chen, Guangjin Pan, Cizhong Jiang, Duanqing Pei, Hongjie Yao

**Affiliations:** 1 Key Laboratory of Regenerative Biology, CAS Center for Excellence in Molecular Cell Science, South China Institute for Stem Cell Biology and Regenerative Medicine, Guangzhou Institutes of Biomedicine and Health, Chinese Academy of Sciences, Guangzhou, 510530, China; 2 Guangdong Provincial Key Laboratory of Stem Cell and Regenerative Medicine, South China Institute for Stem Cell Biology and Regenerative Medicine, Guangzhou Institutes of Biomedicine and Health, Chinese Academy of Sciences, Guangzhou, 510530, China; 3 School of Life Sciences and Technology, Tongji University, Shanghai, 200092, China; 4 Laboratory of RNA Chemical Biology, Guangzhou Institutes of Biomedicine and Health, Guangzhou, 510530, China; 5 Department of Environmental Health Sciences, Johns Hopkins University, Maryland, 21205, USA

## Abstract

Nucleosome positioning and histone modification play a critical role in gene regulation, but their role during reprogramming has not been fully elucidated. Here, we determined the genome-wide nucleosome coverage and histone methylation occupancy in mouse embryonic fibroblasts (MEFs), induced pluripotent stem cells (iPSCs) and pre-iPSCs. We found that nucleosome occupancy increases in promoter regions and decreases in intergenic regions in pre-iPSCs, then recovers to an intermediate level in iPSCs. We also found that nucleosomes in pre-iPSCs are much more phased than those in MEFs and iPSCs. During reprogramming, nucleosome reorganization and histone methylation around transcription start sites (TSSs) are highly coordinated with distinctively transcriptional activities. Bivalent promoters gradually increase, while repressive promoters gradually decrease. High CpG (HCG) promoters of active genes are characterized by nucleosome depletion at TSSs, while low CpG (LCG) promoters exhibit the opposite characteristics. In addition, we show that vitamin C (VC) promotes reorganizations of canonical, H3K4me3- and H3K27me3-modified nucleosomes on specific genes during transition from pre-iPSCs to iPSCs. These data demonstrate that pre-iPSCs have a more open and phased chromatin architecture than that of MEFs and iPSCs. Finally, this study reveals the dynamics and critical roles of nucleosome positioning and chromatin organization in gene regulation during reprogramming.

Reprogramming of MEFs to iPSCs by addition of the Yamanaka factors (Oct4, Sox2, Klf4 and c-Myc) represents a paradigm shift in regenerative medicine^1^. Interestingly, the reprogramming process often results in the production of cells with a stable intermediate ‘pre-iPSC’ state. Pre-iPSCs exhibit morphology identical to that of iPSCs and embryonic stem cells (ESCs) but do not express endogenous pluripotency markers^2^. Moreover, some of these pre-iPSCs can be converted to fully reprogrammed iPSCs by treating them with small molecule inhibitors such as ReproSox or VC^3^,^4^. Thus, MEFs, pre-iPSCs and iPSCs are not only the starting, intermediate and final stages of reprogramming, but also represent useful tools for delineating the molecular changes associated with reprogramming and changes in cell fate^3^.

During the progression from MEFs to iPSCs, the cell undergoes a remodeling of chromatin architecture. Nucleosomes are the fundamental structural units of chromatin, consisting of approximately 146 base pairs (bp) of DNA wrapping around a histone octamer^5^,^6^. Key epigenetic modifications of chromatin structure include the positioning of nucleosomes and covalent modifications to histone tails, which regulate the expression of specific genes^7^,^8^,^9^. Global maps of nucleosome positions in *C. elegans*^10^ *S. cerevisiae*^11^,^12^ and humans^13^,^14^ show that nucleosomes are not randomly distributed throughout the genome but are highly organized, particularly at TSSs. Critically, dynamic changes of nucleosome positions have also been observed during the differentiation of mouse ESCs to neural progenitors^15^. However, understanding how nucleosome positioning and remodeling affects somatic cell reprogramming remains to be determined.

Here, we generated genome-wide maps of nucleosome occupancy and the distributions of active (H3K4me3) and repressive (H3K27me3 and H3K9me3) histone modifications. We investigated these marks during the conversion of MEFs to pre-iPSCs, and then to iPSCs via chromatin immunoprecipitation followed by deep sequencing (ChIP-Seq) and carried out RNA-sequencing (RNA-Seq) to determine transcript abundance. Our study showed that iPSCs possess a more open chromatin architecture than that of MEFs. However, pre-iPSCs display the most open chromatin state among the three cell lines. In addition, nucleosomes in pre-iPSCs are more phased than those in MEFs and iPSCs. Furthermore, cell type-specific genetic programs gradually shuts down, and pluripotency programs are activated. Nucleosome reorganization and histone methylations around TSSs are highly coordinated with gene activities during reprogramming. Bivalent promoters gradually increase and repressive promoters gradually decrease when reprogramming MEFs to pre-iPSCs, then to iPSCs. Active HCG promoters are characterized with nucleosome depletion at TSSs, while LCG promoters feature an abundance of nucleosomes. Furthermore, we show that VC promotes nucleosome reorganization on specific genes during the fate transition from pre-iPSCs to iPSCs. Our results provide insights into the regulation of nucleosome repositioning and provide a basis for studying cell fate transitions during reprogramming.

## Results

### Nucleosome occupancy is reorganized during somatic cell reprogramming

**Figure 1:**
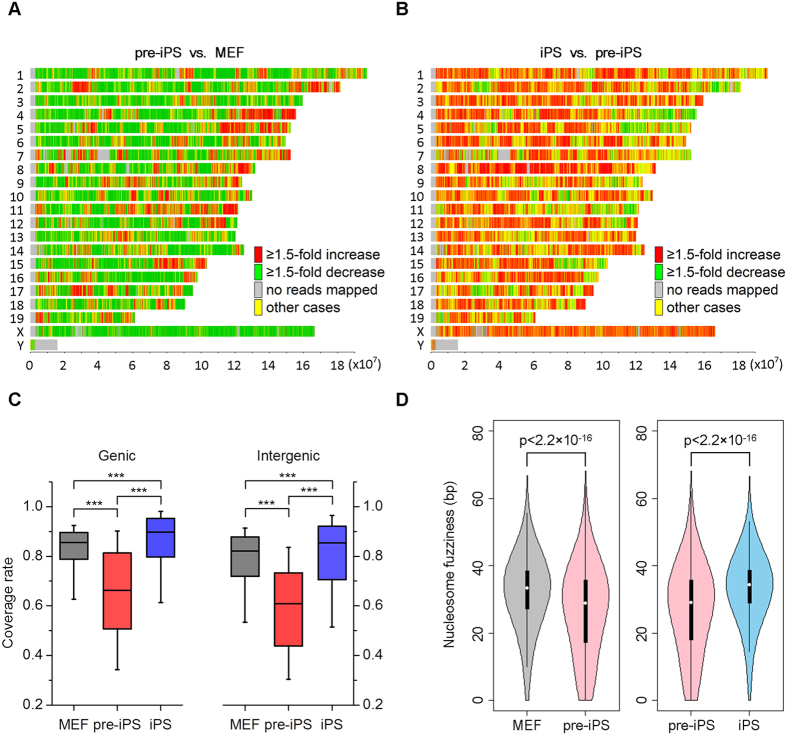
Dynamics of nucleosome organization during somatic cell reprogramming. (**A**) Genome-wide comparison of nucleosome occupancy for pre-iPSCs vs. MEFs. Colours indicate the change in nucleosome occupancy in each 10 kb region between two samples. Red indicates a ≥ 1.5-fold nucleosome occupancy increase in pre-iPSCs, green indicates a ≥1.5-fold nucleosome occupancy decrease in pre-iPSCs, grey indicates no nucleosomes detected, and yellow indicates regions with a <1.5-fold change in nucleosome occupancy. (**B**) Genome-wide comparison of nucleosome occupancy between iPSCs vs. pre-iPSCs as in (**A**). Red indicates a ≥1.5-fold nucleosome occupancy increase in pre-iPSCs, green indicates a ≥1.5-fold nucleosome occupancy decrease in pre-iPSCs, grey indicates no nucleosomes detected, and yellow indicates regions with a <1.5-fold change in nucleosome occupancy. (**C**) Boxplot showing nucleosome coverage rates for both genic and intergenic regions among MEF, pre-iPSCs and iPSCs (***p < 2.2 × 10^−16^). (**D**) Violin-plot showing pair-wise comparison of nucleosome fuzziness among MEF, pre-iPSCs and iPSCs. At left: nucleosome fuzziness distribution for common nucleosomes in pre-iPSCs and MEFs. At right: distribution of common nucleosomes in pre-iPSCs and iPSCs. Nucleosome fuzziness was calculated as the standard deviation of tag locations around the nucleosome dyad. Common nucleosomes between pre-iPSCs/MEFs or iPSCs/pre-iPSCs are defined as those when the distance between two nucleosome dyad locations is less than 73 bp (approximately half the size of nucleosome). Two-tailed and paired t-test was used to evaluate whether the observed difference between two samples was significant.

To characterize the dynamic of epigenomic state during reprogramming, mononucleosomes were isolated from MEFs, pre-iPSCs and iPSCs by digestion with micrococcal nuclease (MNase) and specifically enriched with anti-histone H3/H3K4me3/H3K27me3/H3K9me3 antibodies then subjected to high-throughput sequencing (see details in methods and Supplementary Table S1). A comparison of genome-wide nucleosome occupancy and histone modification among biological replicates indicates a high consistency between samples (Pearson correlation coefficient >0.98; Supplementary Fig. S1), indicative of high data quality. Further, we compared genome-wide nucleosome occupancy in a pairwise manner between MEFs, pre-iPSCs and iPSCs. From MEFs to pre-iPSCs, only 17.32% of the genome was occupied by more nucleosomes and 52.71% of the genome displayed less nucleosome occupancy, suggesting that the genome tend towards nucleosomes loss and a more open chromatin architecture during this stage of reprogramming (Fig. 1A). When pre-iPSCs was induced to iPSCs, nearly half of genomic regions tend to gain nucleosomes and become less open during iPS maturation (Fig. 1B and Supplementary Fig. S2B), suggesting a less open chromatin architecture in iPSCs compared to pre-iPSCs. However, the open chromatin architecture of ES and iPS cells has been documented by numerous researches^16^,^17^,^18^,^19^,^20^,^21^. To explain this paradox, we compared the nucleosome map between MEFs and iPSCs. In support with this view, we found that the whole genome was less occupied by nucleosomes in iPSCs (Supplementary Fig. S2A and S2B), implying an open chromatin state in iPSCs compared to MEFs.

To further elucidate the dynamics of nucleosome deposition during reprogramming, we grouped the genome into genic and intergenic regions. Surprisingly, we found that both genic and intergenic regions of pre-iPSCs showed a lower nucleosome coverage rate than that of MEFs and iPSCs (Fig. 1C). An increase of nucleosome occupancy at not only TSSs and transcription termination sites (TTSs) but also genic regions and correspondingly significant decrease at intergenic regions in pre-iPSCs compared with MEFs or iPSCs was observed when scanning nucleosome distribution in more detailed regions (Supplementary Fig. S2C).

We next calculated nucleosome fuzziness using the standard deviation of tag locations around the nucleosome dyad, and analyzed the nucleosome fuzziness distribution in MEFs, pre-iPSCs and iPSCs. The fuzziness distribution in pre-iPSCs is quite different from that in MEFs and iPSCs (Supplementary Fig. S2D). We extracted and compared common nucleosomes between pre-iPSCs/MEFs and iPSCs/pre-iPSCs when the distance between the nucleosome dyad was less than 73 bp (approximately half the size of nucleosome), and observed that nucleosome fuzziness in pre-iPSCs decreases significantly (Fig. 1D). The average nucleosome fuzziness in pre-iPSCs is 26 bp, much lower than those in both MEFs (31 bp) and iPSCs (32 bp), indicating that nucleosomes showing less deviation in pre-iPSCs are thus increasingly phased. Overall, we concluded that chromatin structures tend to show lower nucleosome occupancy but exhibit more defined nucleosome positioning in pre-iPSCs, which then recovers to more relaxed nucleosome positioning in the fully reprogrammed iPSCs.

### Coordinate transcriptome regulation and nucleosome reorganization are essential for somatic cell reprogramming

**Figure 2:**
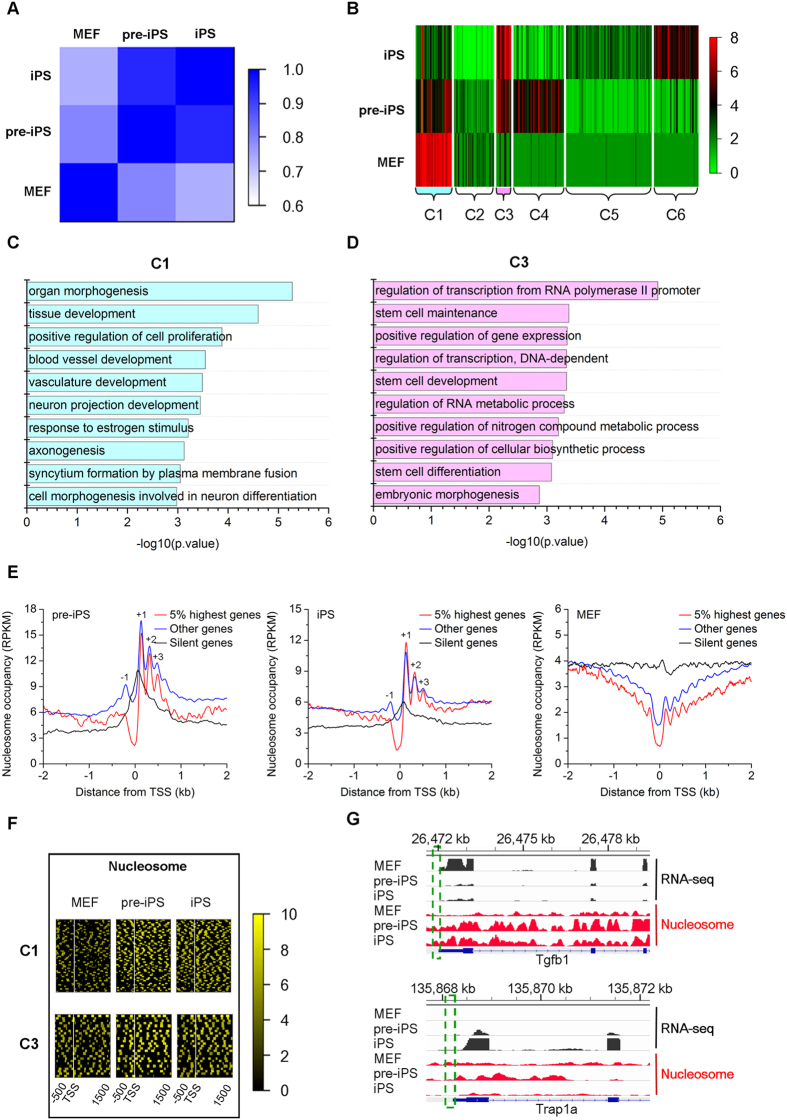
Correlations between gene expression and chromatin state during somatic cell reprogramming. (**A**) Heatmap shows the Pearson correlation of gene transcript profiling of MEFs, pre-iPSCs and iPSCs. (**B**) DE genes were grouped into six clusters by K-means clustering. Transcriptional levels of the clustered DE genes in three cell types are shown as log_2_ (FPKM + 1). (**C**) Biological process ontology of DE genes enriched in cluster 1 (C1) of Fig. 2B. (**D**) Biological process ontology of (**D,E**) genes enriched in cluster 3 (C3) of Fig. 2B. (**E**) Average nucleosome occupancy patterns near TSSs of the 5% highest expressed genes, silent genes and other genes in MEFs, pre-iPSCs and iPSCs. (**F**) Heatmaps show the enrichment of nucleosome signals around the TSSs (indicated by vertical white lines) of DE genes in C1 (top) and C3 (bottom). Each row represents a (−500 bp −1500 bp) TSS region. Genes are ranked by H3K4me3 signal in iPSCs TSS regions. (**G**) Browser tracks (vertical axes-tag count for nucleosome occupancy and gene expression) for a downregulated gene (*Tgfb1*) and upregulated gene (*Trap1a*) obtained by H3 MNase ChIP-seq, and RNA-seq. As indicated in the green dotted boxes, nucleosome occupancy around the TSS increases in the downregulated gene Tgfb1 and decrease clearly in the upregulated gene Trap1a during reprogramming.

To assess how the transcriptome is affected by nucleosome positioning during the stepwise reprogramming process, we carried out an RNA-seq analysis (Supplementary Table S2). Correlation analysis of gene expression profiles indicates high consistency between replicates (Pearson correlation coefficient >0.95; Supplementary Fig. S3). Via Pearson correlation assay, we observed a gradual decrease in the correlation of gene expression between MEFs and reprogrammed cells and an increase in the correlation of gene expression between pre-iPSCs and iPSCs (Fig. 2A). ESCs have the ability to grow indefinitely while maintaining pluripotency and to differentiate into cells of all three germ layers^22^,^23^. We profiled gene expression and nucleosome distribution near the TSSs of pluripotency and germ layer marker genes (Supplementary Fig. S4 and Supplementary Table S3). In contrast to MEFs and pre-iPSCs, nucleosomes at TSSs of pluripotent marker genes are relatively well organized in iPSCs (Supplementary Fig. S4A). Correspondingly, the expression levels of pluripotent marker genes are markedly high, while marker genes for three germ layers are gradually silenced during somatic cell reprogramming (Supplementary Fig. S4B). Specifically, the mesodermal maker genes that are highly expressed in mesoderm-derived MEFs exhibit lower transcriptional activity after reprogrammed to pre-iPSCs, suggesting that nucleosome reorganization occurs during reprogramming.

To more comprehensively illustrate the nucleosome dynamics during reprogramming, we identified 411 differentially expressed (DE) genes in our dataset and grouped these genes into six clusters that show different expression patterns during reprogramming (Fig. 2B and Supplementary Table S4). Genes continuously repressed in C1 are involved in developmental processes such as organ morphogenesis and TGF-β-mediated tissue development (Fig. 2C)^24^,^25^ suggesting that silencing lineage-specific developmental programs is a prerequisite for successful reprogramming. The gradually activated genes in C3 are related to stemness and transcriptional regulation (Fig. 2D), suggesting the activation of a pluripotency program during reprogramming. We also noted that genes in C4 are upregulated during the first stage and then downregulated during iPSCs maturation and that this cluster of genes is mainly involved in epithelial development and cell migration (Supplementary Fig. S5A), in accordance with the observation that a mesenchymal-to-epithelial transition program is launched in MEFs to initiate the early phase of reprogramming^26^. Genes in C6 are repressed at the first stage and activated at the second stage. These genes are mainly involved in gamete generation (Supplementary Fig. S5B), suggesting their importance for the establishment of pluripotency. For example, *Dppa3* was recently reported to be critical for the generation of fully reprogrammed iPSCs, while its depletion resulted in reprogramming to only the pre-iPS state^27^. Despite the differential expression levels among MEFs, pre-iPSCs and iPSCs, expression of C2 and C5 genes is relatively low in these three cell types (Fig. 2B).

Previous studies have shown that nucleosome positioning around TSSs correlates with gene activity. As expected, nucleosome-free regions exist at the TSSs of active genes but not silent genes, and nucleosome depletion become more significant for the most highly expressed genes (Fig. 2E)^15^,^28^. An array of well-positioned nucleosomes (from −1 to + 3) flanking TSSs of active genes are present in both pre-iPSCs and iPSCs (Fig. 2E). Surprisingly, we found that the nucleosome occupancy pattern in MEFs is distinct from that in pre-iPSCs and iPSCs (Fig. 2E). Thus, we further compared the genome-wide nucleosome occupancy data for MEFs and iPSCs from our study with that reported in previous studies^15^,^14^. Correlation analysis at the genome scale indicated high consistency between our data and published data (Supplementary Fig. S6). In addition, we examined the correlation between histone modification pattern and gene activity. As expected, H3K4me3 signals exhibit a strong preference for active promoters, while H3K27me3 and H3K9me3 modifications show negative correlations with the gene expression level (Supplementary Fig. S7). We analyzed these chromatin features around the TSSs of DE genes and observed a coordinated change in both nucleosome positioning and histone modification that was accompanied by the regulation of gene expression during reprogramming (Fig. 2F and Supplementary Figs. S5C and S5D). For example, *Tgfb1*, a C1 gene, was gradually silenced with a concomitant gradual increase of nucleosome occupancy around TSSs, while *Trap1a*, a C3 gene, displayed the opposite trend during reprogramming (Fig. 2G). These results further demonstrate that nucleosomes around TSSs are reorganized during somatic cell reprogramming.

To further dissect the relationship between nucleosome occupancy and gene activity during reprogramming, we grouped the nucleosome occupancy profiles into four clusters according to the similarity of nucleosome occupancy profiles around TSSs based on iPSCs. We observed a dramatic change in nucleosome occupancy around TSSs when MEFs were reprogrammed to pre-iPSCs, and then to iPSCs. We found that a large number of genes occupied by nucleosomes at TSSs are mainly involved in limb morphogenesis, organ development, ion transport and detection of stimulus (top cluster separated by the horizontal white line, Supplementary Fig. S5E). These results are consistent with the above observations that genes related to embryonic development are repressed during reprogramming and that the TSSs of inactive genes tend to be occupied by nucleosomes.

### Chromatin domains are dynamically altered during somatic cell reprogramming

**Figure 3:**
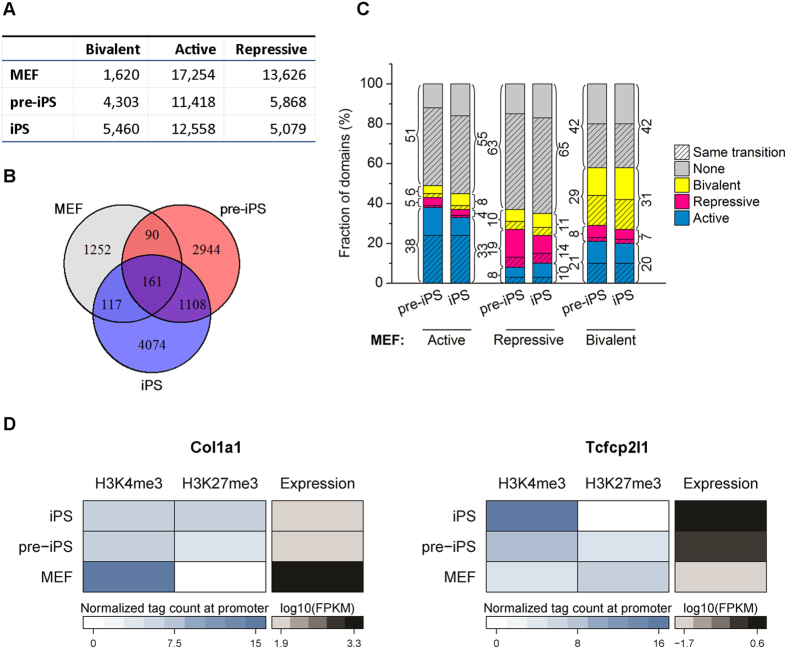
Bivalent, active and repressive domains are cell type-specific during somatic cell reprogramming. (**A**) The number of active, repressive and bivalent domains in MEFs, pre-iPSCs and iPSCs. (**B**) Venn diagram shows the overlap of bivalent domains in the three cell stages. Less than 10% of the bivalent domains are common among MEFs, pre-iPSCs and iPSCs. Most bivalent domains are formed during reprogramming. (**C**) Domains in MEFs (indicated at bottom) are changed to other states during reprogramming. The detailed proportion for each category is labelled. The same transitions from MEFs to pre-iPSCs and to iPSCs are shaded. (**D**) Mesodermal marker gene *Col1a1* located within an active domain in MEFs, is silenced by a bivalent domain in iPSCs (left). Pluripotent marker gene *Tcfcp2l1* located within a repressive domain in MEFs is activated by an active domain in iPSCs (right).

To further investigate chromatin state dynamics during somatic cell reprogramming, we classified active (H3K4me3), repressed (H3K27me3) and bivalent (H3K4me3/H3K27me3) chromatin domains in MEFs, then analyzed the dynamics of these domains when MEFs were induced to pre-iPSCs and then iPSCs. We observed a gradual increase of bivalent domains and a corresponding decrease of repressive domains during reprogramming (Fig. 3A). A large number of bivalent domains are cell type specific and common bivalent domains among MEFs, pre-iPSCs and iPSCs only account for 3–10% (Fig. 3B), suggesting that the histone modification of these domains was altered during reprogramming. To obtain insight into these alternations, we tracked the histone modifications of these domains defined in MEFs during reprogramming to pre-iPSCs and iPSCs. Interestingly, we found that the majority of active domains in MEFs either remain active or lost H3K4me3 and H3K27me3, and only a small portion were changed to either repressive or bivalent domains in pre-iPSCs and iPSCs (Fig. 3C). However, repressive and bivalent domains in MEFs had a tendency to convert to one of other three states during reprogramming (only 19% and 14% of repressive domain in MEFs remained repressive in pre-iPSCs and iPSCs, respectively; only 29% and 31% of bivalent domain in MEFs remained bivalent in pre-iPSCs and iPSCs, respectively) (Fig. 3C). Furthermore, changes in chromatin domains were accompanied by changes in gene expression. For example, the mesodermal marker gene *Col1a1* was located in an active domain in MEFs but was silenced and in a bivalent domain in pre-iPSCs and iPSCs (Fig. 3D). Simultaneously, the pluripotent marker gene *Tcfcp2l1* was located in a repressive domain and silenced in MEFs was activated in pre-iPSCs and iPSCs by an active domain (Fig. 3D).

### Dynamics of H3K4me3/H3K27me3 on HCG and LCG promoters during somatic cell reprogramming

**Figure 4:**
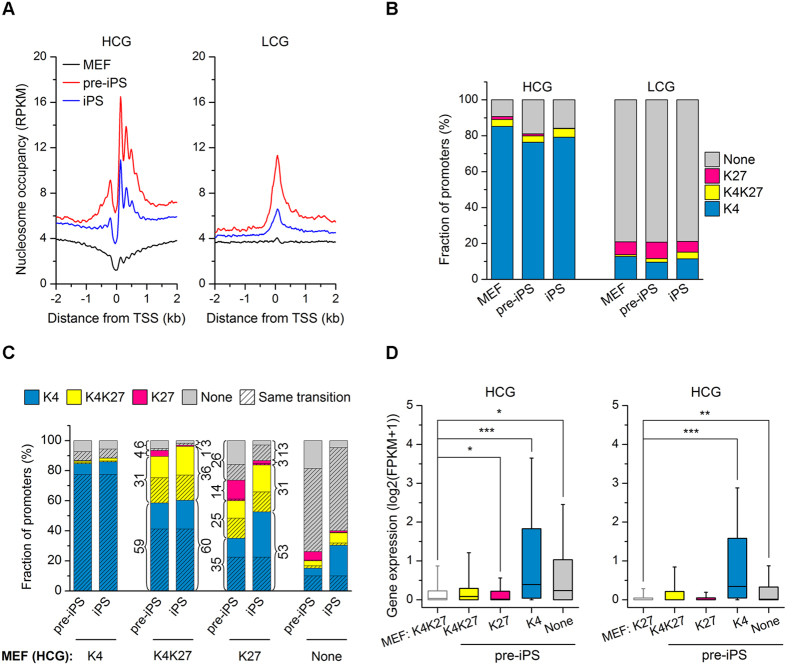
Dynamics of H3K4 and H3K27 histone trimethylation at HCG and LCG promoters during reprogramming. (**A**) Average nucleosome occupancy pattern around HCG and LCG promoters in MEFs, pre-iPSCs and iPSCs. (**B**) Most HCG promoters are marked by H3K4me3 (blue), while most LCG promoters exhibit neither mark (grey) in all three cell types. (**C**) H3K4me3 and H3K27me3 status at HCG promoters in pre-iPSCs and iPSCs are dependent on their status in MEFs (indicated at the bottom). The detailed proportion for each state in pre-iPSCs and iPSCs is indicated for the “K4K27” and “K27” states in MEFs. The transition from MEFs to pre-iPSCs and to iPSCs is shaded. HCG promoters marked by H3K4me3 only in MEFs tend to retain this mark, while promoters marked by neither mark tend to retain their original state. Promoters marked by H3K4me3 and H3K27me3 tend to lose one or both marks, while promoters marked by only H3K27me3 tend to convert to one of the other three states. (**D**) Expression levels of HCG genes marked by both H3K4me3 and H3K27me3 (left) or by H3K27me3 alone (right) change with the chromatin state during reprogramming. from MEFs to pre-iPSCs. Statistical significance, two-tailed t-test. From K4K27 to other states (left panel), *p = 0.011 (to K27), ***p < 2.2 × 10^−16^ (to K4), *p = 0.014 (to None). From K27 to other states (right panel), ***p < 2.2 × 10^−16^ (to K4), *p = 0.0098 (to None).

Previous results have indicated that CpG islands are primed to be promoters by default in mammals and that HCG and LCG promoters can be distinguished by their DNA sequence composition^29^. We examined the relationships between nucleosome occupancies of HCG and LCG promoters and gene expression during reprogramming. A significant nucleosome occupancy peak at TSSs was observed at LCG promoters (Fig. 4A), similar to the nucleosome occupancy pattern of silent genes (Fig. 2E). In contrast, HCG promoters were characterized by a nucleosome-depleted region at TSSs and high nucleosome enrichment in the flanking regions (Fig. 4A), similar to the pattern observed for active genes (Fig. 2E). Correspondingly, genes with HCG promoters tended to be active, while genes with LCG promoters tended to be silent (Supplementary Fig. S8A). Most HCG promoters were marked by H3K4me3, but not by H3K27me3. In contrast, neither H3K4me3 nor H3K27me3 was detected at a majority of LCG promoters (Fig. 4B).

These results led us to speculate that the dynamic loading of histone modifications onto HCG and LCG promoters may occur during reprogramming. To address this question, we investigated the dynamics of histone H3K4me3 and H3K27me3 on HCG and LCG promoters in MEFs, pre-iPSCs and iPSCs (Fig. 4C and Supplementary Figs. S8B and S8C). For HCG promoters, most active promoters marked by H3K4me3 in MEFs remained active in pre-iPSCs and iPSCs (Fig. 4C). The promoters marked by both H3K4me3 and H3K27me3 in MEFs tended to lose H3K27me3 and become active promoters in pre-iPSCs and iPSCs (Fig. 4C). In addition, HCG promoters modified by H3K27me3 alone in MEFs tended to convert to one of the other three states (H3K4me3 alone, H3K4me3/H3K27me3, or none) in pre-iPSCs and iPSCs (Fig. 4C). The changes of H3K4me3/H3K27me3 at LCG promoters were similar to those at HCG promoters (Fig. 4C and Supplementary Fig. S8B). However, the active mark H3K4me3 at LCG promoters was markedly reduced compared to that at HCG promoters (Fig. 4C and Supplementary Fig. S8B). In addition, we observed highly similar dynamics of active promoters from MEFs to pre-iPSCs or to iPSCs, but much lower similarity for bivalent and repressive promoters (Fig. 4C and Supplementary Fig. S8B).

Though HCG and LCG promoters exhibit distinct gene activities, dynamic changes of epigenetic features at both HCG and LCG promoters during reprogramming could alter gene expression. HCG promoters with bivalent or repressive marks in MEFs are transcriptionally activated in pre-iPSCs when they are marked by the active mark H3K4me3 alone, or continue to be repressed when they retain bivalent or repressive marks (Fig. 4D). We also observed changes in gene expression accompanying changes of various histone modifications during reprogramming (Supplementary Fig. S8C). Thus, we conclude that the activities of HCG and LCG promoters are dynamically regulated by their histone modification status.

### VC promotes nucleosome reorganization during transition from pre-iPSCs to iPSCs

**Figure 5:**
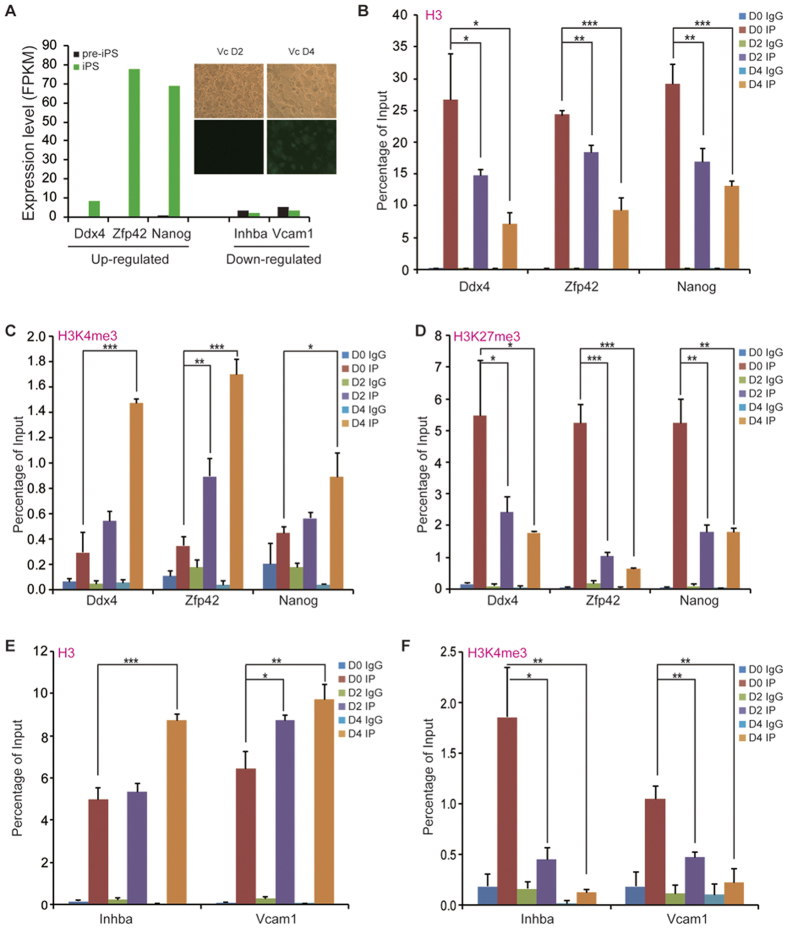
VC promotes the reorganization of histone H3 as well as H3K4me3- and H3K27me3-modified nucleosomes during transition from pre-iPSCs to iPSCs. (**A**) Induction of iPSCs from pre-iPSCs by treatment with VC and Setdb1 siRNA. Cells were first treated with Setdb1 siRNA, and grown in mES medium supplemented with VC for 48 h and 96 h, respectively. RNA-seq data show the upregulated (*Ddx4*, *Zfp42* and *Nanog*) and downregulated genes (*Inhba* and *Vcam1*) during the pre-iPS to iPS transition. (**B**) Dynamics of nucleosome deposition at the *Ddx4*, *Zfp42* and *Nanog* promoters, quantified by ChIP-qPCR in pre-iPSCs cultured with mES medium containing VC. *Ddx4* H3 ChIP (D2 vs. D0 *p = 0.0462, D4 vs. D0 *p = 0.0102); *Zfp42* H3 ChIP (D2 vs. D0 **p = 0.0018, D4 vs. D0 ***p = 0.0002); *Nanog* H3 ChIP (D2 vs. D0 **p = 0.0050, D4 vs. D0 ***p = 0.0010). (**C**) Dynamics of H3K4me3 at the *Ddx4*, *Zfp42* and *Nanog* promoters, quantified by ChIP-qPCR in pre-iPSCs cultured with mES medium containing VC. *Ddx4* H3K4me3 ChIP (D4 vs. D0 ***p = 0.0002); *Zfp42* H3K4me3 ChIP (D2 vs. D0 **p = 0.0041, D4 vs. D0 ***p = 7.86 × 10^−5^); *Nanog* H3K4me3 ChIP (D4 vs. D0 *p = 0.0249). (**D**) Dynamics of H3K27me3 at the *Ddx4*, *Zfp42* and *Nanog* promoters, quantified by ChIP-qPCR in pre-iPSCs cultured with mES medium containing VC. *Ddx4* H3K27me3 ChIP (D2 vs. D0 *p = 0.0432, D4 vs. D0 *p = 0.0215); *Zfp42* H3K27me3 ChIP (D2 ***p = 0.0003, D4 vs. D0 ***p = 0.0002); *Nanog* H3K27me3 ChIP (D2 vs. D0 **p = 0.0017, D4 vs. D0 **p = 0.0015). (**E**) Dynamics of nucleosome deposition at the *Inhba* and *Vcam1* promoters, quantified by ChIP-qPCR in pre-iPSCs cultured with mES medium containing VC. *Inhba* H3 ChIP (D4 vs. D0 ***p = 0.0005); *Vcam1* H3 ChIP (D2 vs. D0 *p = 0.0108, D4 vs. D0 **p = 0.0071). (**F**) Dynamics of H3K4me3 at the *Inhba* and *Vcam1* promoters, quantified by ChIP-qPCR in pre-iPSCs cultured with mES medium containing VC. *Inhba* H3K4me3 ChIP (D2 vs. D0 *p = 0.0140, D4 vs. D0 **p = 0.0068); *Vcam1* H3K4me3 ChIP (D2 vs. D0 **p = 0.0022, D4 vs. D0 **p = 0.0017). Data represent the mean ± s.d. of triplicates.

We recently demonstrated that VC participates in the transition from pre-iPSCs to iPSCs, and knockdown of Setdb1 accelerates this process^3^,^30^. Pre-iPSCs treated with VC and Setdb1 siRNA yield OCT4-GFP clones at day 4 after treatment, indicating that pre-iPSCs were converted to iPSCs (Fig. 5A). We were curious to know whether VC plays the role in nucleosome positioning during the transition from pre-iPSCs to iPSCs. Therefore, we analyzed our RNA-Seq data and selectively tested three upregulated genes (*Zfp42*, *Ddx4* and *Nanog*) and two downregulated genes (*Inhba* and *Vcam1*) in iPSCs compared with those in pre-iPSCs (Fig. 5A) to further investigate the recruitment of histone H3, H3K4me3 and H3K27me3 to the promoters of these genes during the transition from pre-iPSCs to iPSCs (Fig. 5B–F). All three upregulated genes are important for ESC pluripotency and reprogramming, while the two down-regulated genes are related to developmental processes^31^,^32^. For example, *Inhba* is a TGF-beta family member gene and participates in the TGF-beta signaling pathway^31^. Notably, ChIP-qPCR results for histone H3, H3K4me3 and H3K27me3 at day 0, 2 and 4 after treatment of pre-iPSCs with VC and *Setdb1* siRNA indicate that the upregulated genes show nucleosome depletion at their promoter regions (such as *Zfp42*, *Ddx4* and *Nanog*), while the downregulated genes display the opposite trend during the transition from pre-iPSCs to iPSCs (Fig. 5B,E). Consistent with H3K4me3 as an active mark and H3K27me3 as a repressive mark, we observed increased levels of H3K4me3 and decreased levels of H3K27me3 at these upregulated genes, as well as decreased levels of H3K4me3 at the downregulated genes (Fig. 5C,D,F). However, we did not observe a significant increase in H3K27me3 at downregulated genes (data not shown), suggesting that other repressive histone marks may play a role in silencing *Inhba* and *Vcam1*. These results suggest that VC regulates nucleosome reorganization and histone modification during cell fate conversion from pre-iPSCs to iPSCs.

## Discussion

Dramatic chromatin reconfiguration occurs during the conversion of somatic cells to iPSCs^16^,^17^. To investigate the role of nucleosome positioning and histone methylation in somatic cell reprogramming, we analyzed high-resolution maps for nucleosome positioning and typical histone modifications (H3K4me3, H3K27me3 and H3K9me3), and further characterized the changes in these epigenetic features during reprogramming. Previous studies have argued that iPSCs and ESCs possess relatively open chromatin structures compared with those of differentiated cells^34^. Here we propose that a large number of nucleosomes first disassemble and then reassemble during reprogramming, and that intermediate pre-iPSCs are characterized by comparatively more open chromatin structure and highly phased nucleosome positioning, suggesting a nucleosome remodeling roadmap to the pluripotency state. Recently, Andras Nagy and colleagues characterized genomic changes, including histone modifications and DNA methylations as well as transcriptomic alternations during reprogramming^34^,^35^,^36^,^37^,^38^. They characterized a new category of pluripotent cells (F-class) that do not exhibit an intermediate state of reprogramming. We therefore compared the transcriptome profiles of MEFs, pre-iPSCs, iPSCs and F-class cells, and found that the transcription profile of F-class cells can be distinguished easily from those of the cell types during reprogramming (Supplementary Fig. S9). Furthermore, we compared the epigenetic state of intermediate pre-iPSCs and F-class cells. As expected, our pre-iPSCs show distinct H3K4me3 and H3K27me3 state from those of F-class cells (Supplementary Fig. S9). These observations confirmed that intermediate pre-iPSCs are distinct, both transcriptionally and epigenetically, from F-class cells.

Active and silent genes exhibit distinct nucleosome occupancy patterns. Although many factors can contribute to the regulation of gene expression, we observed a strong correlation between nucleosome positioning and gene expression, indicating that the flexibility of gene expression control is partially affected by the nucleosome modulation. Nucleosome depletion at TSSs may be required for the binding of transcription factors, thereby facilitating transcriptional activation. However, nucleosome occupancy at the TSSs of silent genes blocks DNA access. Successful reprogramming of MEFs to iPSCs is accompanied by the activation of pluripotency genes and the repression of lineage-specific genes. As expected, marker genes for the three germ layers were gradually repressed and their TSSs exhibited nucleosome occupancy in iPSCs, while pluripotency genes gradually showed a nucleosome occupancy pattern similar to that of active genes. These results suggest extensive nucleosome remodeling, concomitant to the process of reprogramming.

Histone modification also plays important roles in regulation of reprogramming. Active genes usually exhibit well-organized nucleosome phasing as well as high H3K4me3 signal at their promoters. The presence of both the active mark H3K4me3 and repressive mark H3K27me3 provides another layer of regulatory code. These bivalent domains are enriched in pluripotent stem cells and may repress gene transcription to poise genes for rapid activation when differentiation is induced^39^,^40^. In support of this idea, we observed an increase in bivalent domains and a corresponding decrease of both active and repressive domains during reprogramming. We also found that almost all of these domains are highly cell type-specific, suggesting that genes marked by bivalent domains tend to be poised for activation in iPSCs.

Regulation of gene expression during reprogramming is collaboratively controlled by nucleosome occupancy, histone modification and DNA sequence. Local chromatin state regulates the accessibility to transcription factors, coregulators, and other components of the basic transcriptional machinery to specific DNA elements in target genes, thus, the dynamic modulation of the chromatin microenvironment determines specific transcriptional programs^41^. For somatic cells to successfully reprogram to iPSCs, collaborative modulation of epigenetic regulators such as histone deacetylases, H3K27 demethylase (Utx) and H3K9 demethylases are required to extensively remodel epigenetic markers across the genome. In this study, we observed that dynamic change in chromatin state (i.e., H3K4me3 and H3K27me3 levels) at both HCG and LCG promoters, accompanied by gene expression regulation is required to convert somatic cells to iPSCs. These results demonstrate the participation of epigenetic modifiers in reprogramming^42^,^43^,^44^.

VC promotes the generation of iPSCs by regulating either histone demethylase Jhdm1a/1b or DNA demethylase TET1^45^,^46^. In addition, VC directly converts pre-iPSCs to iPSCs by modulating H3K9 methylation^3^. In this study, we found that VC promotes nucleosome reorganization at the promoters of both pluripotency genes and developmental genes, which may facilitate the conversion of pre-iPSCs to iPSCs. The binding of Foxa2 to nucleosomal DNA on H2A.Z-containing nucleosomes promotes nucleosome disassembly complexes (Nap1l1-coupled SWI/SNF and INO80) to enable nucleosome depletion during ES cell differentiation into endoderm^47^. It will be interesting to investigate the chromatin remodeling factors that bring about the depletion of nucleosomes in pre-iPSCs and promote the more rigid positioning of nucleosomes during the intermediate stage of somatic cell reprogramming.

In summary, we provided a nucleosome remodeling roadmap from MEFs to iPSCs and conclude that the stage-specific organizations of nucleosomes and histone modifications represent an additional regulatory process that controls DNA access by regulatory factors to manipulate genes expression during somatic cell reprogramming. Our study strengthens our understanding of the reprogramming process, which in turn benefits our understanding of cellular development and differentiation. We are sure that nucleosome positioning and histone modification function to turn on or off the temporally and spatially specific genes to keep the individual’s identify at different stages of development or differentiation^15^,^48^. Furthermore pathogenic processes can affect the cell-fate transition and it is also becoming clear that epigenetic changes are associated with disease states^49^,^50^,^51^. The new picture of nucleosome reorganization should help us to understand new insights into the mechanisms involved in gene regulation and human diseases. Therefore, our insights into the orchestration of reprogramming to pluripotency by nucleosome repositioning could elucidate developmental and pathological processes, potentially leading to new therapeutic applications^41^.

## Methods

### Ethic Statement

*Mus musculus* were maintained and cared for in our Experimental Animal Centre’s facility, in accordance with Guangzhou Institutes of Biomedicine and Health Institutional Animal Care and Use Committee (approve number 2013024) protocols.

### Cell culture

MEFs were derived from day 13.5 mouse embryos from crossing male Oct-GFP transgenic allele-carrying mice (CBA/CaJ × C57BL/6J) to 129Sv/Jae female mice. Pre-iPSCs and iPSCs were induced from this kind of MEFs by using four Yamanaka factors. MEFs were maintained in fibroblast medium: DMEM supplemented with 10% fetal bovine serum (FBS), 1% L-glutamine, and 1% non-essential amino acid (NEAA). Two stable clones of pre-iPSCs and their corresponding iPSCs used in this study were obtained from our previous report^3^. These pre-iPSCs and iPSCs were cultured and expanded with mES medium (DMEM supplemented with 15% FBS, 1% Glutamax, 1% NEAA, 1% sodium pyruvate, 0.1 mM 2-mercaptoentanol, leukaemia inhibitory factor (lif)) on a MEF-derived feeder layer. VC was purchased from Sigma. The working concentration of VC was 50 μg/ml.

### Mononucleosome preparation

Cells were crosslinked with 1% formaldehyde for 10 min at room temperature. After quenching crosslinking with 125 mM glycine for 5 min, cells were washed twice with ice-cold PBS containing 1 mM PMSF and 1 × EDTA-free protease inhibitor cocktails. Mononucleosomes were prepared according to SimpleChIP^**®**^ Enzymatic Chromatin IP protocols from Cell Signaling Technology. Briefly, for isolation of mononucleosomes from pre-iPSCs and iPSCs, 4 × 10^7^ crosslinked cells were resuspended in 10 ml buffer A containing 1 mM DTT, 1 × protease inhibitor cocktails and 1 mM PMSF, and incubated on ice for 10 min, mixed by inverting tube every 3 min. Nuclei lysates were centrifuged at 3,000 rpm at 4 °C for 5 min and then resuspended in 10 ml ice-cold buffer B containing 1 mM DTT. The nuclei was collected and resuspended in 1 ml buffer B containing 1 mM DTT for MNase digestion. 8 μl of MNase (Cell Signaling Technology, #10011 S) was added to the nuclei and incubated at 37 °C for 25 min. While for isolation of mononucleosomes from MEFs, 3 × 10^7^ cells were crosslinked and nuclei was isolated. Then 16 μl of MNase was added to the nuclei and incubated at 37 °C for 10 min. Enzyme digestion was quenched by adding a final concentration of 50 mM EDTA and sonication was performed for 11 cycles to solubilize nucleosomes. Then nucleosomes were centrifuged at 10,000 rpm at 4 °C for 10 min and the supernatant was saved for later ChIP experiments.

### ChIP-Seq

About 18 μg mononucleosomes were incubated with 10 μg antibodies (Details of antibodies are listed in Supplementary Table S5) for each immunoprecipitation overnight at 4 °C. 30 μl protein G dynabeads were then added for 2 hr at 4 °C with rotation. Immune complexes were washed with low salt wash buffer three times and high salt wash buffer once. Mononucleosomes were eluted twice from the protein G magnetic beads and reverse crosslinked with 0.2 M NaCl at 65 °C overnight and digested protein with protease kinase (PK) afterwards. Then, the ChIPed DNA was purified with MinElute PCR purification Kits (Qiagen) and quantitated by Quant-iT^TM^ Picogreen^®^ dsDNA reagent (Invitrogen). The ChIP-Seq libraries were constructed following the Illumina ChIP-Seq library generation protocol. Briefly, 10 ng ChIP DNA was blunt-ended, and then a dA tail was added. Illumina genomic adaptors with index sequences were ligated to the DNA. The adaptor-ligated DNA was amplified by PCR for 18 cycles. Then DNA was diluted to 10 nM before sequencing. Sequencing on a HiSeq2000 was carried out by Beijing Genomics Institutes (BGI) in Shenzhen and Ribobio in Guangzhou.

### ChIP-Seq reads mapping

ChIP-Seq reads were aligned to the mouse mm9 reference genome using Bowtie (version 1.0.1)^52^. Only uniquely aligned reads with no more than two mismatches were used for the subsequent analysis. For chromatin features (nucleosome and histone modifications), the aligned reads were then shifted 73 bp (about half of the nucleosome size) in the 3’ direction to reach their dyad positions. A cutoff was set to keep duplicate reads (the same coordination and the same strand) such that at least 99% uniquely aligned reads were remained.

### Comparison of genome-wide distribution of chromatin features between replicates

The whole genome was divided into 100-kb regions and the ChIP-Seq reads (nucleosome/H3K4me3/H3K27me3) was counted in each genomic region and then normalized as Reads Per Kilobase per Million mapped reads (RPKM). The logarithm values (base 10) of nucleosome/H3K4me3/H3K27me3 occupancy (RPKM) in all 100-kb genomic regions were visualized by a scatter plot for each pair of replicates. Consistency degree of genome-wide distribution of chromatin features was measured by Pearson correlation coefficient.

### Genome-wide comparison of nucleosome occupancy

For each chromosome, nucleosome read counts were binned in 10 kb intervals and divided by the total number of uniquely mapped reads. Comparisons between samples were conducted bin-by-bin for each chromosome. Different colors were used to represent change levels of nucleosome occupancy between two samples, red indicates a ≥1.5-fold increase, green indicates a ≥1.5-fold decrease, grey indicates the absence of detected nucleosomes, and yellow indicates all other cases.

### Chromatin features in genic and intergenic regions

The coverage rate was calculated as the fraction of each region that is covered by nucleosomes using BEDTools software (version 2.16.2)^53^. The nucleosome coverage rate was calculated for each region of at least 150 bp in length. Nucleosome distribution across the genome was further explored by calculating the percentage of nucleosome reads in more detailed regions (i.e., 300 bp upstream of TSSs, 600 bp downstream of TSSs, 300 bp upstream of TTSs, 300 bp downstream of TTSs, and the remaining genic and intergenic regions).

### Average occupancy profile of chromatin features around TSSs

A UCSC mouse genome annotation file (mm9) was used to define TSSs. The number of ChIP-Seq reads mapping to each site (between 2 kb upstream and 2 kb downstream of each TSS) was counted and summed according to their relative distance from corresponding TSSs across all genes. For comparison, the read distributions around TSSs were normalized as RPKM values. Smoothing was conducted for distribution plots by 10 bp-interval binning with a 5-bin moving average and 1-bin step size.

### Heatmaps of chromatin feature distribution around TSSs

Locations of nucleosomes, including histone-modified nucleosomes, were predicted by GeneTrack (version 1.0.3)^54^. Nucleosomes located near TSSs (500 bp upstream to 1000 bp downstream) were extended from the dyad position to a variable length upstream and downstream determined by the fuzziness of the corresponding nucleosome. Nucleosome fuzziness was measured as the standard deviation of the read distribution for the nucleosome. Genes were clustered by K-means clustering according to the similarity of occupancy profiles in a variable region. To characterize nucleosome occupancy at all genes or at lineage-specific genes, nucleosome distribution patterns were grouped into four clusters by K-means clustering according to the similarity of nucleosome occupancy profiles at regions 300 bp upstream to 600 bp downstream of a TSS. The same gene order was used for MEFs and pre-iPSCs. To characterize the chromatin feature distribution around TSSs of clustered DE genes, genes were ranked by H3K4me3 signal in the region 300 bp upstream to 600 bp downstream of TSSs in iPSCs. The same gene order was used for MEFs and pre-iPSCs.

### Analysis of bivalent, active and repressive domains

Domains were defined by a method used in a previous study, with slight modification^55^. We calculated reads mapping to each 1000 bp window across the genome and scanned the windows containing at least six reads. Then, we grouped consecutive windows falling above this threshold, allowing a gap of one sub-threshold window. Grouped windows with at least 60 H3K4me3 or H3K27me3 ChIP-seq reads were defined as H3K4me3 or H3K27me3 islands. Bivalent domains were defined by a ≥50% overlap of H3K4me3 and H3K27me3 islands. Active domains are defined as H3K4me3 islands that do not overlap with H3K27me3 islands, and repressive domains are H3K27me3 islands that do not overlap with H3K4me3 islands. For comparisons between samples, two domains with at least 50% overlap were considered common domains.

### Analysis of the dynamic chromatin state of HCG and LCG promoters

We defined a promoter as the region spanning 500 bp upstream to 500 bp downstream of a TSS. HCG promoters were defined by overlap with CpG islands^56^. CpG island data were downloaded from the CpG Cluster2 database^57^. The rest of the annotated promoters were classified as LCG promoters. The histone trimethylation states of HCG and LCG promoters were grouped into four classes depending on the H3K4me3 and H3K27me3 levels. A promoter with an H3K4me3 (or H3K27me3) level higher than 1 RPKM was classified as “K4” (or “K27”) state, while a promoter with both H3K4me3 and H3K27me3 levels higher than 1 RPKM was regarded as “K4K27” state. Promoters with both H3K4me3 and H3K27me3 levels less than 1 RPKM were regarded as “None”.

### ChIP-qPCR

ChIP-qPCR assays were performed as previously described with some modifications^58^. Briefly, crosslinked cells digested as described above. The digested chromatin supernatant was diluted with ChIP dilution buffer and then co-incubated with antibodies-dynabeads. After immunoprecipitation, the beads was wash with low salt wash buffer once, high salt wash buffer once, LiCl wash buffer once and TE buffer twice and then the DNA was reverse-crosslinked and purified for qPCR analysis. Primers used for ChIP-qPCR are listed in Supplementary Table S6.

### RNA-Seq

MEFs were maintained and cultured for no more than three passages before preparing for RNA-Seq. Pre-iPSCs and iPSCs were both cultured for 6 passages. Before RNA-Seq, cultured cells were harvested and dissolved in Trizol for total RNA extraction and treated with DNase I (Ambion) to remove any potential contaminated DNA fraction. The following library generation and sequencing were conducted by BGI in Shenzhen.

### RNA-Seq Analysis

Tophat (version 2.0.4)^59^ was used to map the RNA-seq reads to the UCSC mouse genes (mm9). Only the uniquely mapped reads were retained for analysis in our study. Cuffdiff (version 2.0.2)^60^ was employed to calculate the expression abundances measured as Fragment Per Kilobase per Million mapped fragments (FPKM) and identify the DE genes. The consistency degree of gene expression profiles between replicates was measured by Pearson correlation coefficient and visualized by scatter plots. For pairwise comparison among MEFs, pre-iPSCs and iPSCs, Pearson correlation analysis was performed based on gene expression levels (FPKM) and visualized by heatmap. The genes whose expression level changed in the compared two samples are equal to or greater than two folds with a false discovery rate (FDR)-adjusted p-value (denoted as q-value) less than 0.05 are defined as DE genes. We further combined all DE genes from pairwise comparisons and conducted K-means clustering to classify these DE genes into six groups that show different expression patterns during reprogramming. Gene ontology (GO) analysis for each group of these DE genes was conducted using DAVID (version 6.7) gene annotation tool^61^.

### Comparison with published data

MNase-Seq data for MEFs^15^ (GSM1004654) and iPSCs^14^ (SRX273997) were downloaded from the NCBI Gene Expression Omnibus (GEO) and the Short Read Archive (SRA). As with the comparison between replicates, the consistency of between our genome-wide nucleosome occupancy data and the published data were measured by Pearson correlation coefficient and visualized by scatter plots.

Transcript profiles and epigenomic states of intermediate pre-iPSCs were compared with those of recently reported F-class cells^34^. The relationship between pre-IPSCs and F-class cells were explored by hierarchical clustering analysis and principal component analysis (PCA). For epigenomic state comparison, we calculated H3K4me3/H3K27me3 levels in the first exon bin for each transcript and compared these levels with those in F-class cells using PCA based on the correlation matrix.

### Statistical analysis and reproducibility

Experimental data are presented as the mean ± standard deviation (s.d.) as indicated in the figure legends. To evaluate whether the observed difference between two groups was significant, we employed the Student’s *t*-test. A p value < 0.05 was considered statistically significant; *p < 0.05, **p < 0.01, ***p < 0.001. The reproducibility of the experiments was as follows: two biological replicates for both ChIP-Seq (nucleosome, H3k4me3 and H3K27me3) and RNA-Seq, three independent technical repeats (Fig. 5B–F).

## Additional Information

**Accession code**: ChIP-seq and RNA-seq data described in this study has been submitted to the NCBI Gene Expression Omnibus under accession number GSE60627

**How to cite this article**: Huang, K. *et al.* Dynamically reorganized chromatin is the key for the reprogramming of somatic cells to pluripotent cells. *Sci. Rep.* **5**, 17691; doi: 10.1038/srep17691 (2015).

## Supplementary Material

Supplementary Information

Supplementary Tables 1-6
