# Supplementary material for: Dynamically reorganized chromatin is the key for the reprogramming of somatic cells to pluripotent cells
Source: Sci Rep. 2015 Dec 7;5:17691. doi: 10.1038/srep17691 (PMC4671053; doi:10.1038/srep17691)
Supplement: Supplementary Information [file srep17691-s1.doc]

**Supplementary Information**

**Dynamically reorganized chromatin is the key for the reprogramming of somatic cells to pluripotent cells**

Kaimeng Huang^1,2¶^, Xiaobai Zhang^3¶^, Jiejun Shi^3¶^, Mingze Yao^1,2^, Jiannan Lin^3^, Jiao Li^1,2^, He Liu^1,2^, Huanhuan Li^1,2^, Guang Shi^1,2^, Zhibin Wang^5^, Biliang Zhang^4^, Jiekai Chen^1,2^, Guangjin Pan^1,2^, Cizhong Jiang^3^, Duanqing Pei^1,2*^, Hongjie Yao^1,2*^

^1^Key Laboratory of Regenerative Biology, South China Institute for Stem Cell Biology and Regenerative Medicine, Guangzhou Institutes of Biomedicine and Health, Chinese Academy of Sciences, Guangzhou, 510530, China.

^2^Guangdong Provincial Key Laboratory of Stem Cell and Regenerative Medicine, South China Institute for Stem Cell Biology and Regenerative Medicine, Guangzhou Institutes of Biomedicine and Health, Chinese Academy of Sciences, Guangzhou, 510530, China.

^3^School of Life Sciences and Technology, Tongji University, Shanghai, 200092, China.

^4^Laboratory of RNA Chemical Biology, Guangzhou Institutes of Biomedicine and Health, Guangzhou, 510530, China.

^5^Department of Environmental Health Sciences, Johns Hopkins University, Maryland, 21205, USA.


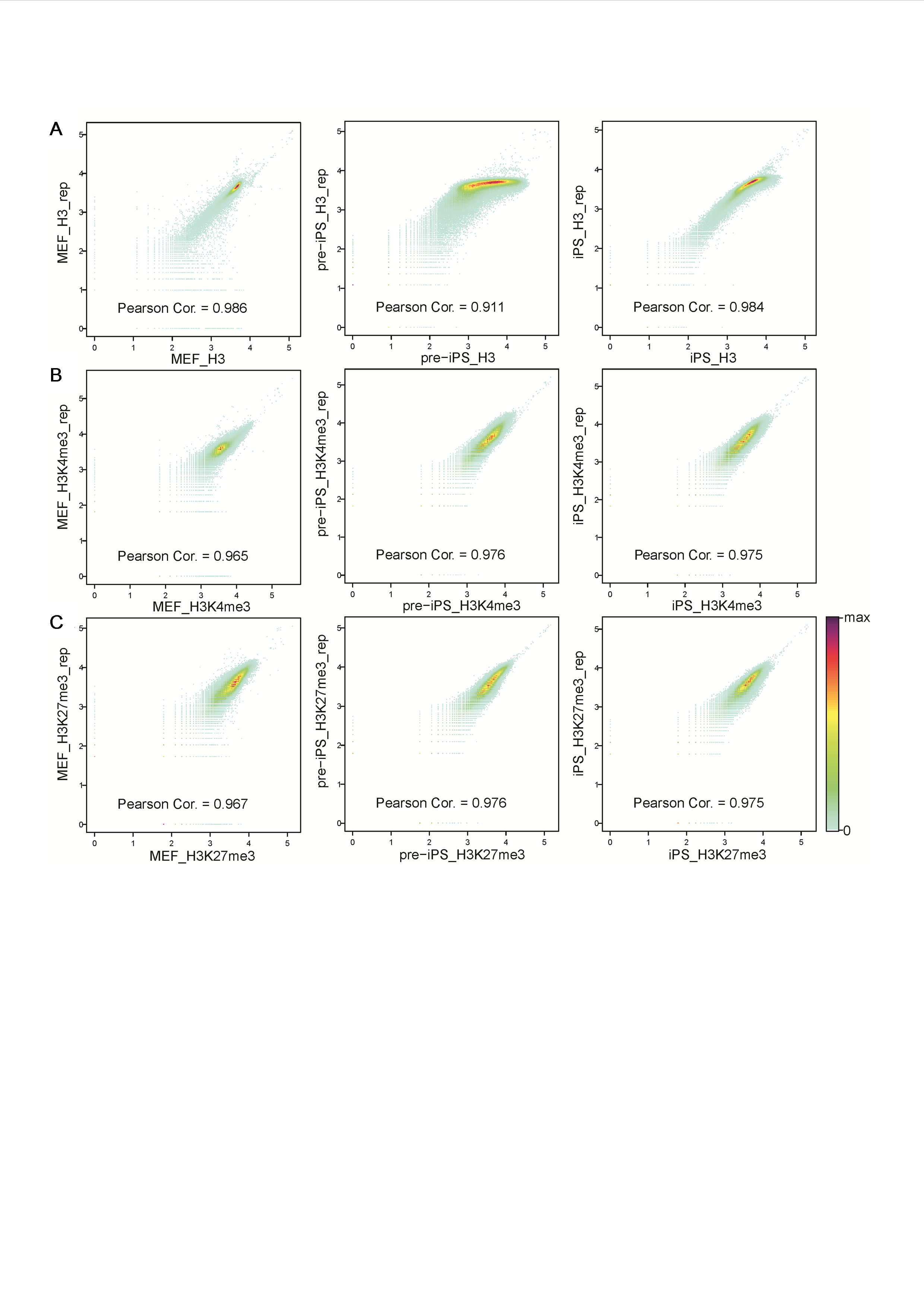


**Fig. S1. Genome-wide comparison of nucleosome occupancy and histone modifications display high consistency of biological replicates.** The whole genome was divided into 10-kb regions and nucleosome/H3K4me3/H3K27me3 occupancy was calculated in each genomic region as RPKM. Each point in scatter plots represents the logarithm value of the nucleosome/H3K4me3/H3K27me3 occupancy in a 10 kb genomic region with base 10 in the two replicates. Colors in scatter plots indicate the spatial density of points from low (light green) to high (dark purple). Pearson correlation coefficient for each pair of replicates was calculated based on the logarithm values of the nucleosome/H3K4me3/H3K27me3 occupancy at the genome-wide scale. (A) Scatter plots show genome-wide comparison of nucleosome occupancy for each pair of replicates. (B) Scatter plots show genome-wide comparison of H3K4me3 occupancy for each pair of replicates. (C) Scatter plots show genome-wide comparison of H3K27me3 occupancy for each pair of replicates.

**
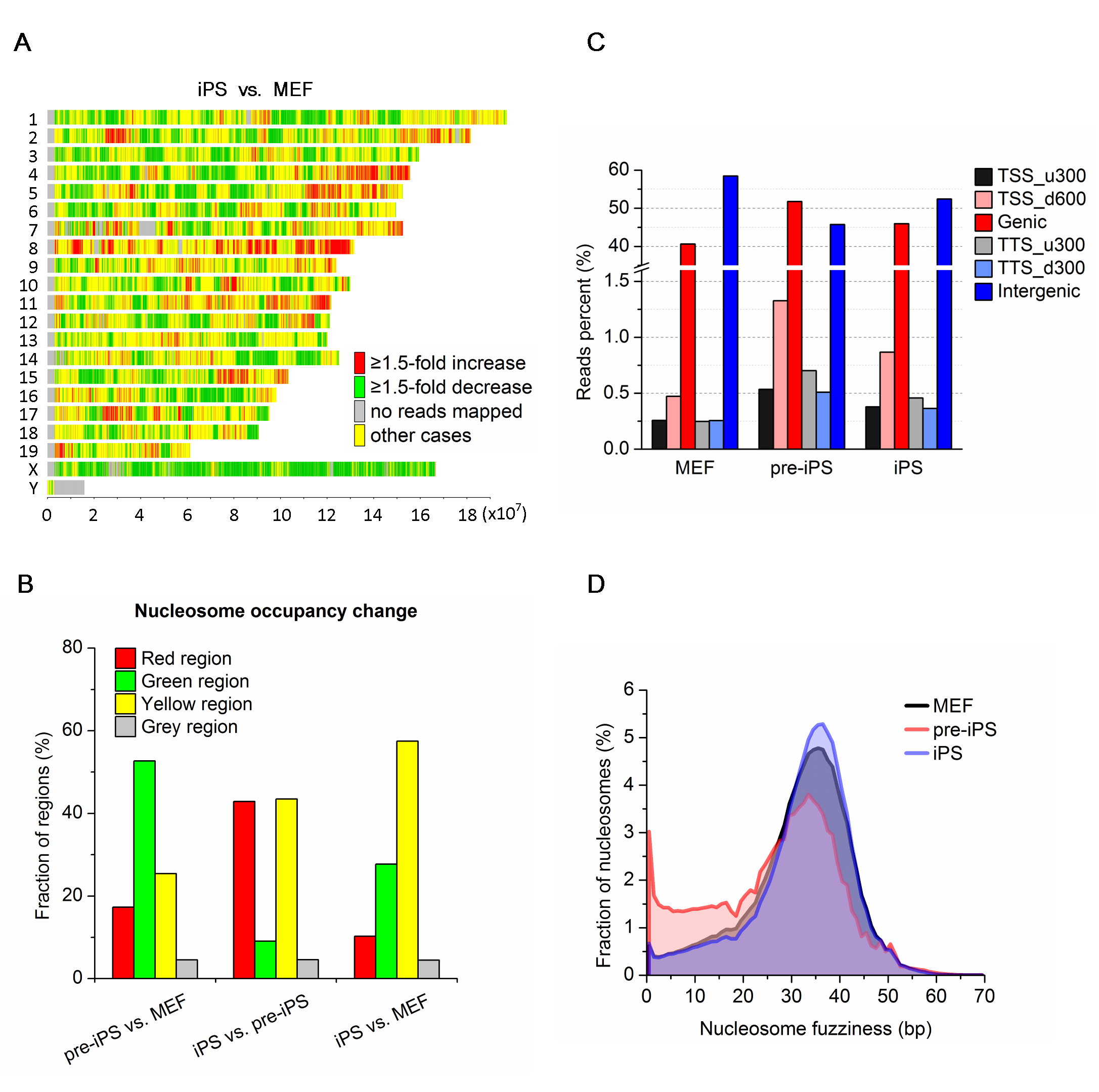
**

**Fig. S2. Genome-wide analysis of nucleosome occupancies in MEFs, pre-iPSCs and iPSCs.** (A) Genome-wide comparison of nucleosome occupancy for iPSCs vs. MEFs. Colors indicate change levels of nucleosome occupancy in each 10 kb region between two samples. Red indicates 1.5 fold or more nucleosome occupancy increase in iPSCs, green indicates 1.5 fold or more nucleosome occupancy decrease in iPSCs, grey indicates no nucleosome detected, and yellow indicates regions with less than 1.5 fold nucleosome occupancy change. (B) Bar plot shows the proportion of different color regions for the pairwise comparison of genome-wide nucleosome occupancy. (C) Nucleosome occupancy decreases in intergenic regions in pre-iPSCs. Bar plot shows the percentage of nucleosome reads located in different genomic regions including 300 bp upstream regions of TSSs (TSS_u300), 600 bp downstream regions of TSSs (TSS_d600), 300 bp upstream regions of TTSs (TTS_u300), 300 bp downstream regions of TTSs (TTS_d300), the rest of genic regions (Genic) and the rest of intergenic regions (Intergenic). (D) Nucleosome fuzziness distribution in MEFs, pre-iPSCs, and iPSCs.

**

**

**Fig. S3. Correlation analysis of gene expression profiles indicates high consistency between replicates for MEFs, pre-iPSCs and iPSCs.** Pearson correlation coefficient for each pair of replicates was calculated based on the logarithm of gene expression with base 2.


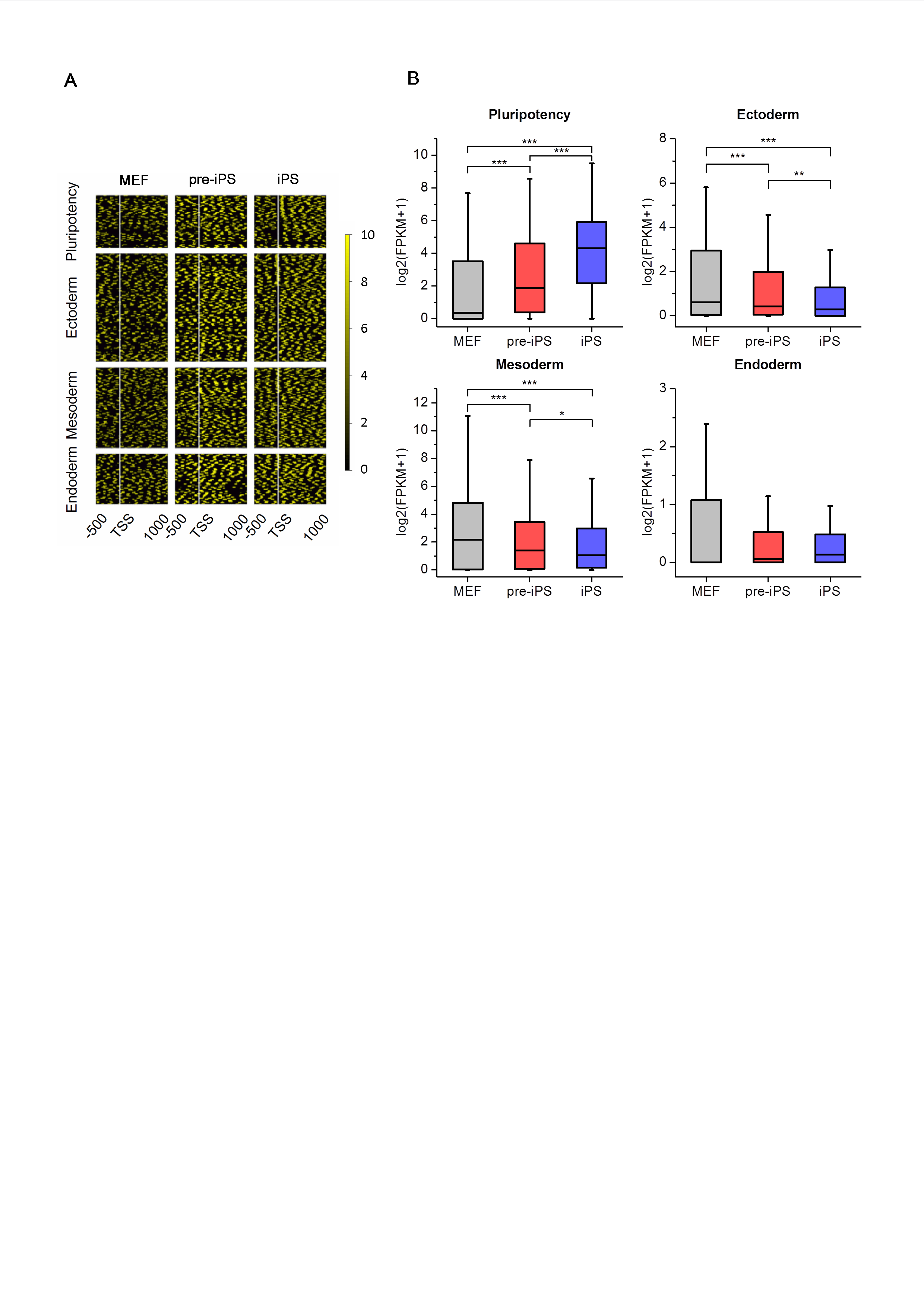


**Fig. S4. Nucleosome distribution and gene expression of lineage-specific marker genes.** (A) Dynamics of nucleosome occupancy of selected lineage-specific marker genes during somatic cell reprogramming. Heatmaps show nucleosome distribution around TSSs of pluripotent, ectodermal, mesodermal and endodermal markers in MEFs, pre-iPSCs and iPSCs. (B) Pluripotent marker genes are activated during reprogramming, while marker genes from three germ layers tend to be repressed. One-tailed and paired t-test was used to detect the statistical significance of gene expression difference. For pluripotent marker genes, MEF vs. pre-iPS *** p=3.774×10^-4^, MEF vs. iPS *** p=1.440×10^-7^, pre-iPS vs. iPS *** p=2.181×10^-4^. For ectodermal marker genes, MEF vs. pre-iPS *** p=1.439×10^-5^, MEF vs. iPS *** p=1.395×10^-6^, pre-iPS vs. iPS ** p=8.395×10^-3^. For mesodermal marker genes, MEF vs. pre-iPS *** p=2.861×10^-5^, MEF vs. iPS *** p=5.794×10^-6^ , pre-iPS vs. iPS * p=0.01004.

**
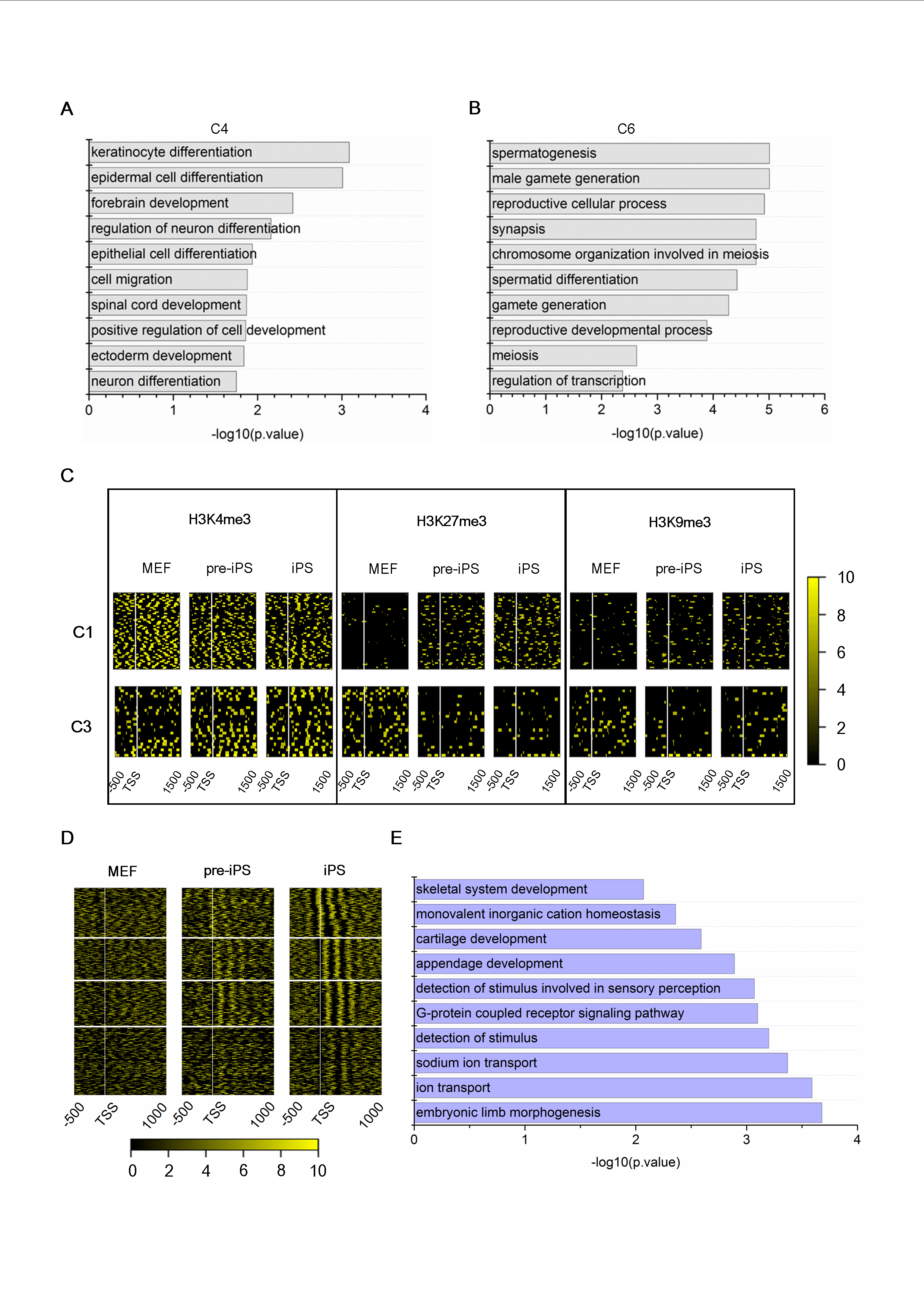
**

**Fig. S5. The correlations between gene expression and chromatin state during somatic cell reprogramming.** (A-B) Enriched biological process of DE genes in C4 (A) and C6 (B) in Figure 2B. (C) Heatmaps show enrichment of H3K4me3, H3K27me3 and H3K9me3 signals around TSSs (indicated by white vertical lines) of DE genes in C1 and C3. Each row represents a (-500 bp to 1500 bp) TSS region. Genes are ranked by H3K4me3 signal in TSS regions in iPSCs. (D) Heatmaps showing nucleosome distribution around all TSSs (indicated by white vertical lines) from 500 bp upstream to 1000 bp downstream. The nucleosome distribution patterns in iPSCs are clustered into four clusters (separated by white horizontal lines) by K-means according to the similarity of nucleosome occupancy profiles in TSS regions from 300 bp upstream to 600 bp downstream. The order is maintained in MEFs and pre-iPSCs to visualize nucleosome occupancy dynamics during reprogramming. (E) Enriched biological process for the genes in the top cluster in Supplementary Fig S5D.


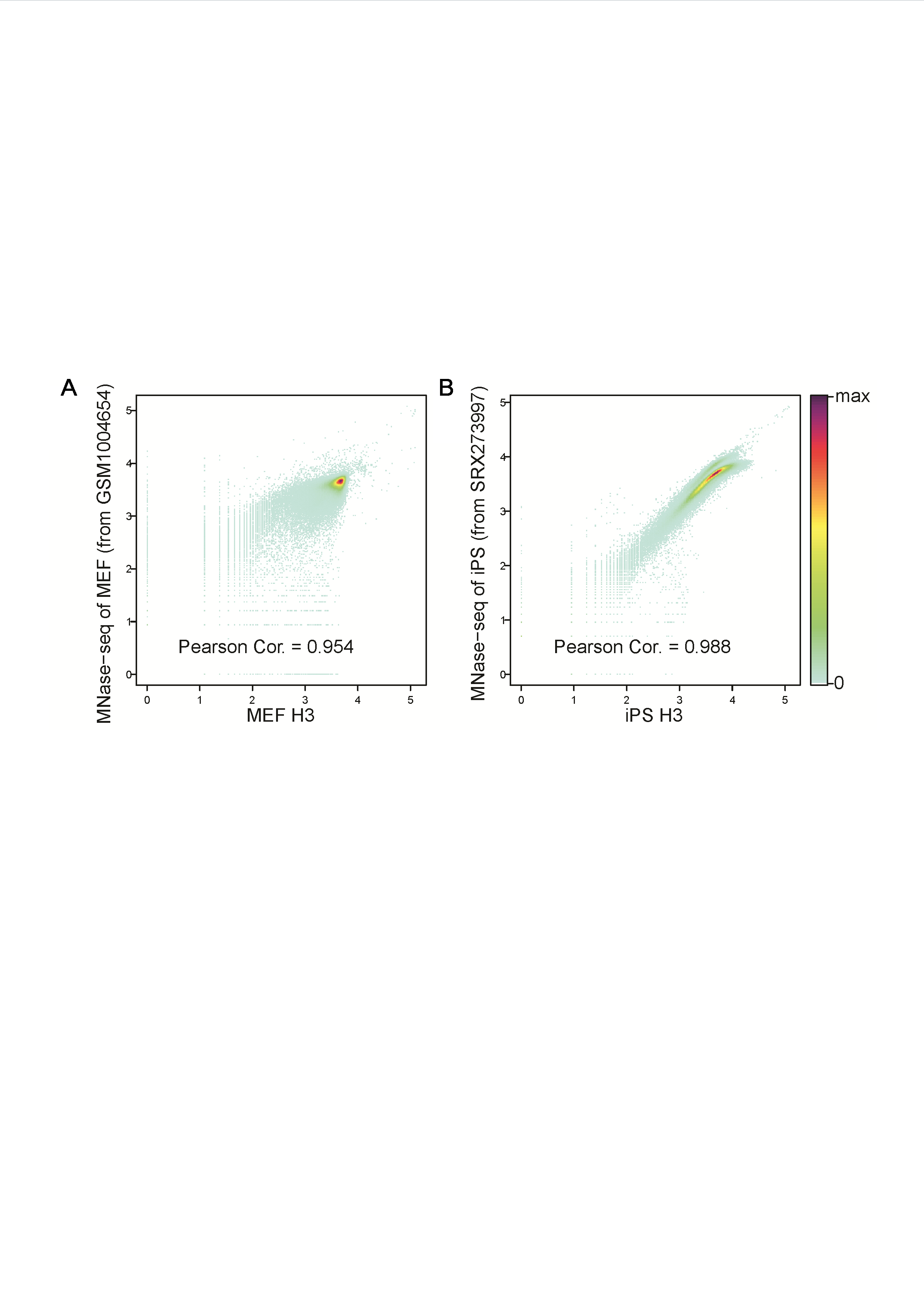


**Fig. S6. Genome-wide comparison of nucleosome occupancy displays high consistency between our data and published data.** The whole genome was divided into 10-kb regions and nucleosome occupancy was calculated in each genomic region as RPKM. Each point in scatter plots represents the logarithm value of the nucleosome occupancy in a 10 kb genomic region with base 10 in the two samples. Colors in scatter plots indicate the spatial density of points from low (light green) to high (dark purple). Pearson correlation coefficient was calculated based on the logarithm values of the nucleosome occupancy at the genome-wide scale.


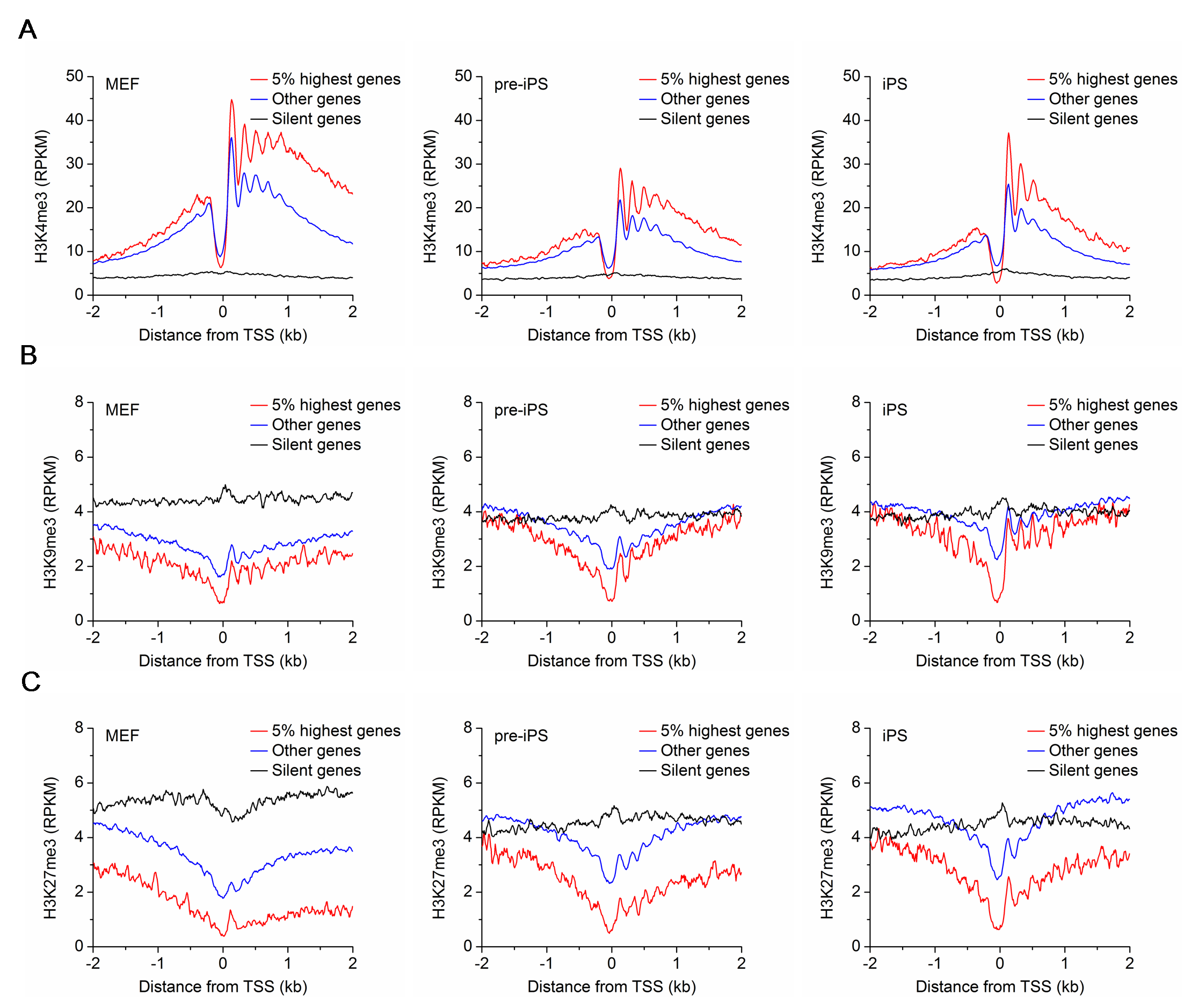


**Fig. S7. Dynamic occupancies of H3K4me3, H3K9me3 and H3K27me3 around TSSs of the 5% most highly expressed genes, silent genes and the other genes in MEFs, pre-iPSCs and iPSCs.** (A) Profiles of H3K4me3 occupancy patterns around TSSs of the 5% most highly expressed genes, silent genes and the other genes in MEFs, pre-iPSCs and iPSCs. (B) Profiles of H3K9me3 occupancy patterns around TSSs of the 5% most highly expressed genes, silent genes and the other genes in MEFs, pre-iPSCs and iPSCs. (C) Profiles of H3K27me3 occupancy patterns around TSSs of the 5% most highly expressed genes, silent genes and the other genes in MEFs, pre-iPSCs and iPSCs.

**
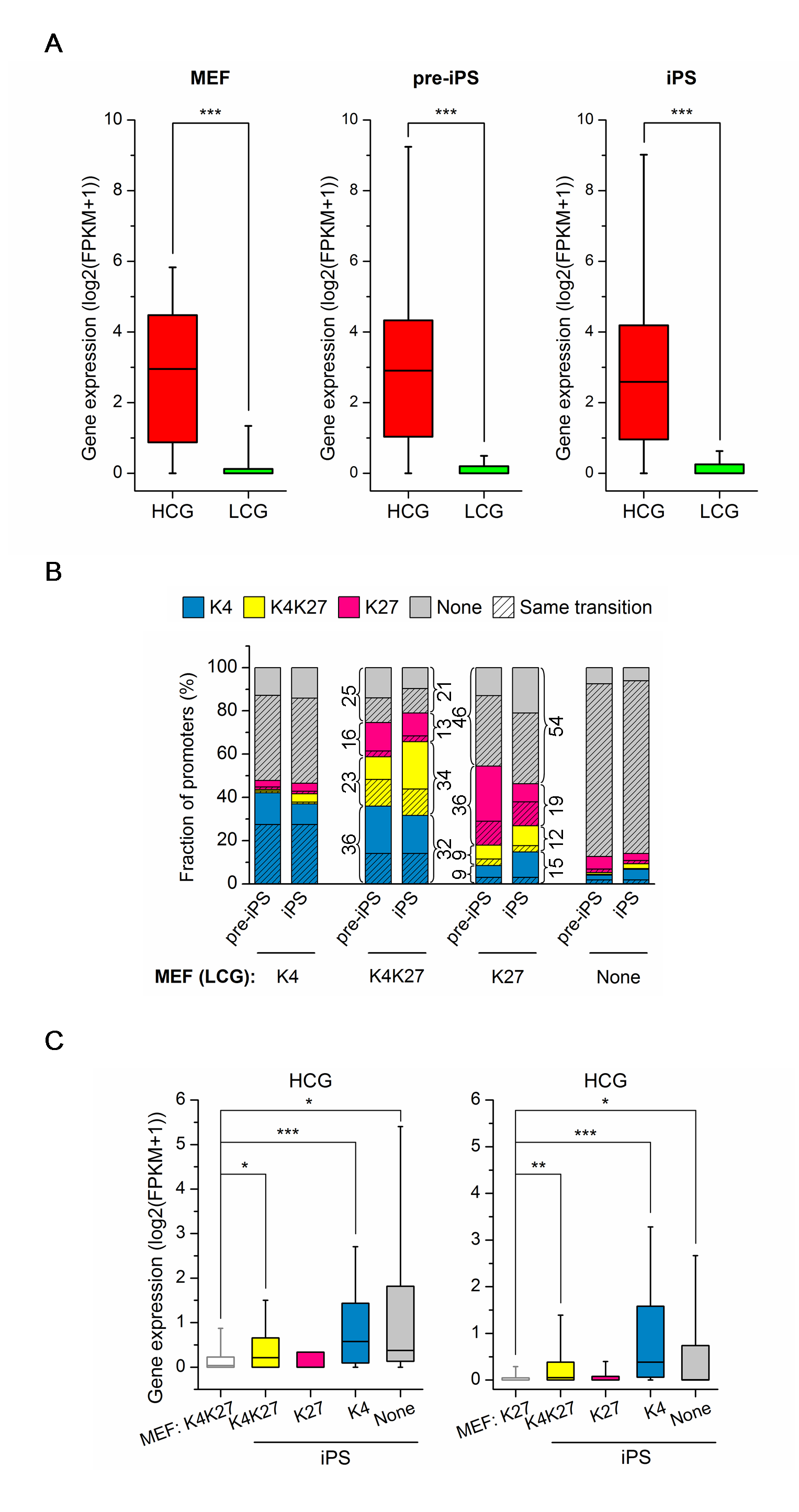
**

**Fig. S8. Dynamics of H3K4me3 and H3K27me3 levels of HCG and LCG promoters during somatic cell reprogramming**. (A) Genes with HCG promoters are much more active than those with LCG promoters in all the three cell types. Statistical significance, one-tailed t-test, HCG vs. LCG, *** p<2.2×10^-16^ (in MEFs), *** p<2.2×10^-16^ (in pre-iPSCs), *** p<2.2×10^-16^ (in iPSCs). (B) H3K4me3 and H3K27me3 state on LCG promoters in pre-iPSCs and iPSCs, conditional on their states in MEFs (indicated at the bottom). Same transition from MEFs to pre-iPSCs and to iPSCs is shaded. Detailed proportion for each state in pre-iPSCs and iPSCs is labeled next to the curly braces for “K4K27” and “K27” state in MEFs. (C) Expression levels of HCG genes marked by both H3K4me3 and H3K27me3 (left) or with H3K27me3 only (right) change with the chromatin state during reprogramming from MEFs to iPSCs. Statistical significance, two-tailed t-test. From K4K27 to other states (left panel), * p=0.0013 (to K4K27), *** p<2.2×10^-16^ (to K4), * p=0.023 (to None). From K27 to other states (right panel), ** p=0.003 (to K4K27), *** p<2.2×10^-16^ (to K4), * p=0.012 (to None).

**
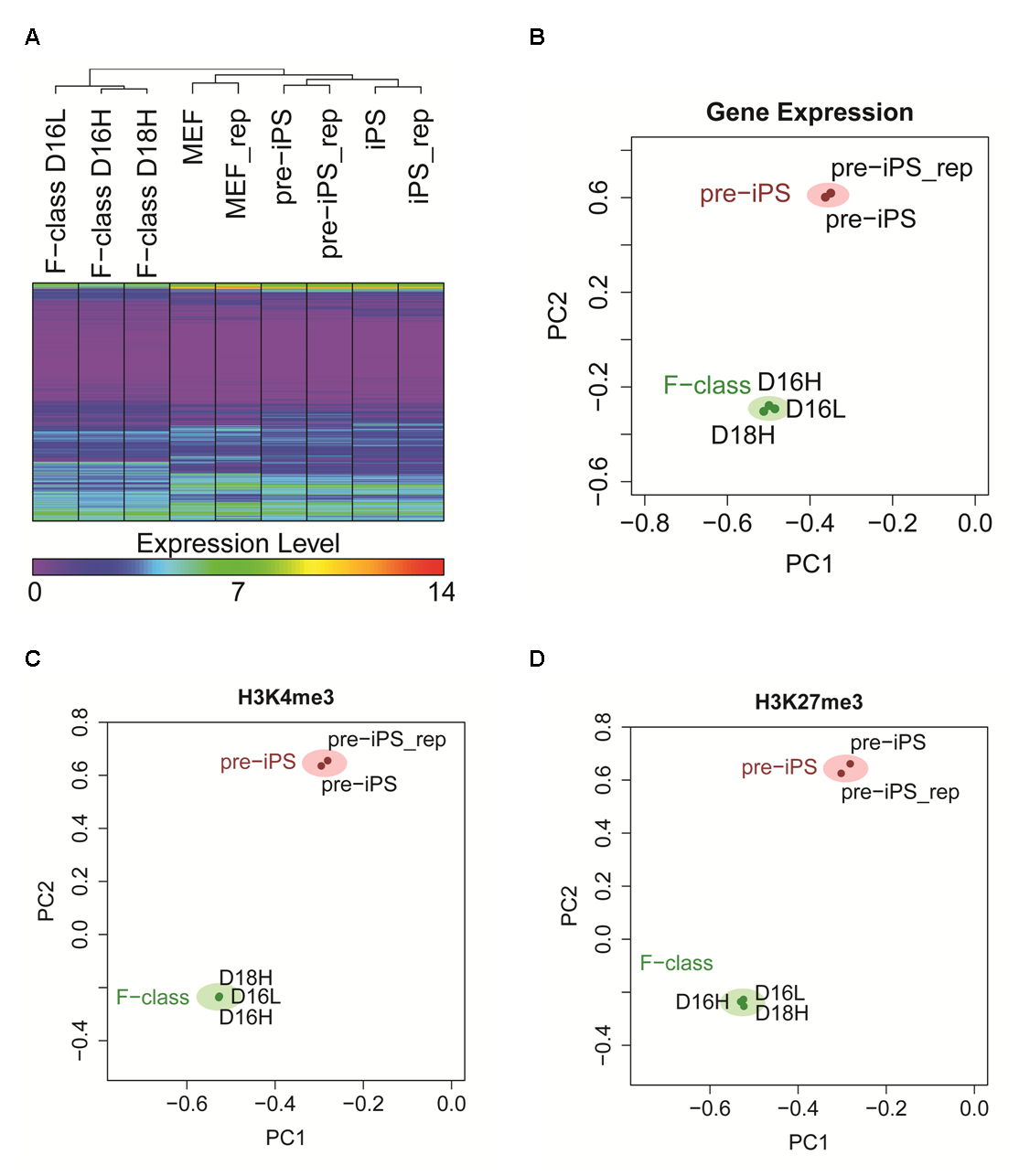
**

**Fig. S9. The intermediate pre-iPSCs of somatic cell reprogramming is distinct from F-class cells.** (A) Hierarchical cluster analysis indicates distinct gene expression profile of F-class cells from those of our cells types. Heatmap shows gene expression profiles visualized by log2(FPKM+1) of each sample. (B) Principal component analysis (PCA) on gene expression profiles shows that our pre-iPSCs can been clearly separated from F-class cells. (C) PCA on H3K4me3 indicates different H3K4me3 state between pre-iPSCs and F-class cells. (D) PCA on H3K27me3 indicates different H3K27me3 state between pre-iPSCs and F-class cells.
